# Biological Evaluation of Triorganotin Derivatives as Potential Anticancer Agents

**DOI:** 10.3390/molecules28093856

**Published:** 2023-05-02

**Authors:** Valeria Stefanizzi, Antonella Minutolo, Elena Valletta, Martina Carlini, Franca M. Cordero, Anna Ranzenigo, Salvatore Pasquale Prete, Daniel Oscar Cicero, Erica Pitti, Greta Petrella, Claudia Matteucci, Francesca Marino-Merlo, Antonio Mastino, Beatrice Macchi

**Affiliations:** 1 Department of Chemical Science and Technology, University of Rome “Tor Vergata”, 00133 Rome, Italy; valeriastefanizzi1995@gmail.com (V.S.); ele.valletta90@gmail.com (E.V.); cicero@scienze.uniroma2.it (D.O.C.); petrella@scienze.uniroma2.it (G.P.); 2 Ph.D. Course in Microbiology, Immunology, Infectious Diseases, and Transplants (MIMIT), University of Rome “Tor Vergata”, 00133 Rome, Italy; 3 Department of Experimental Medicine, University of Rome “Tor Vergata”, 00133 Rome, Italy; antonellaminutolo@gmail.com (A.M.); carlinimartina@libero.it (M.C.); matteucci@med.uniroma2.it (C.M.); 4 Department of Chemistry Ugo Schiff, University of Florence, 50019 Florence, Italy; franca.cordero@unifi.it (F.M.C.); anna.ranzenigo@unifi.it (A.R.); 5 Department of Systems Medicine, University of Rome “Tor Vergata”, 00133 Rome, Italy; salvatore.prete@uniroma2.it; 6 Department of Chemical, Biological, Pharmaceutical, and Environmental Sciences, University of Messina, 98166 Messina, Italy; fmarino@unime.it; 7 The Institute of Translational Pharmacology, CNR, 00133 Rome, Italy; antonio.mastino@ift.cnr.it

**Keywords:** tin, organotin derivatives, cytotoxicity, growth inhibition, tumor cells, cell death, anticancer agents

## Abstract

Metal-derived platinum complexes are widely used to treat solid tumors. However, systemic toxicity and tumor resistance to these drugs encourage further research into similarly effective compounds. Among others, organotin compounds have been shown to inhibit cell growth and induce cell death and autophagy. Nevertheless, the impact of the ligand structure and mechanisms involved in the toxicity of organotin compounds have not been clarified. In the present study, the biological activities of commercially available bis(tributyltin) oxide and tributyltin chloride, in comparison to those of specially synthesized tributyltin trifluoroacetate (TBT-OCOCF_3_) and of cisplatin, were assessed using cells with different levels of tumorigenicity. The results show that tributyltins were more cytotoxic than cisplatin in all the tested cell lines. NMR revealed that this was not related to the interaction with DNA but to the inhibition of glucose uptake into the cells. Moreover, highly tumorigenic cells were less susceptible than nontumorigenic cells to the nonunique pattern of death induced by TBT-OCOCF_3_. Nevertheless, tumorigenic cells became sensitive when cotreated with wortmannin and TBT-OCOCF_3_, although no concomitant induction of autophagy by the compound was detected. Thus, TBT-OCOCF_3_ might be the prototype of a family of potential anticancer agents.

## 1. Introduction

The success of *cis*-diaminedichloroplatinum (II) (cisplatin) and of the second-generation derivatives carboplatin and oxaliplatin has opened a new perspective for the development of metal-based drugs for the treatment of several solid tumors [1]. Although this class of chemotherapeutic agents has a broad anticancer spectrum and is also used in combination, their systemic toxicity and tumor drug resistance have encouraged ongoing research for new metal-based drugs. Over the years, organotin compounds (i.e., nonplatinum but tin-containing substances) have been investigated in different models as potential anticancer drug candidates [2]. Originally, organotin compounds, formed by linking tin directly with an organic substituent, were generally known for their environmental toxicity and used as biocides, fungicides, and pesticides, and to preserve paints for marine vessels [3]. Over the past century, several organotin derivatives have been synthesized and tested in vitro and in vivo for their toxicities [4,5,6] and anticancer activities [7]. Several organotin derivatives were designed by variations of the organic moieties and donor ligands linked to the metal to obtain more antiproliferative compounds [8]. This approach has given rise to several diorganotin and triorganotin (IV) compounds tested in different in vitro models with various results.

In particular, tributyltin (TBT) compounds were shown to induce cell death through different mechanisms. Exposure of cortical neurons to TBT chloride (TBT-Cl) reduced the phosphorylation of the mammalian target of rapamycin (mTOR), triggering cell death preceded by autophagy [9]. Treatment of Jurkat cells with bis(tributylin) oxide (TBT-O) induced reticulum endoplasmic stress followed by NF-kB and T-cell activation and apoptosis [10]. Both TBT-Cl and triphenyltin (TPT) chloride (TPT-Cl) increased BAX expression in human estrogen and a receptor-positive breast adenocarcinoma MCF-7 cell line [11]. In particular, TBT-Cl stimulated p53 expression and BCL-2 downregulation in tumor cells more than TPT-Cl, thus presumably influencing the route towards regulated cell death (RCD) [11].

More recently, several structure-based approaches have been taken to improve organotin compounds’ cytotoxic and potential anticancer activities. Several structures involving diorganotin and carboxylate, carbamate, amide, thiolate, and dithiocarbamate ligands improved stability and water solubility. Dibutyltin(IV) compounds induced cell death independently of p53 [12]. Tribenzyltin carboxylates complexes were shown to induce caspase activation and morphological changes related to apoptosis in the MCF-7, MDA-MB231, and 4T1 cell lines [13], while TBT(IV) carboxylates induced selective cytotoxicity versus the THP-1 and HEP-2G cell lines [14]. Di- and triphenyltin(IV) dithiocarbamates were shown to be cytotoxic and antiproliferative towards an erythroleukemia cell line [2]. Exposure of human NT2/D1 embryonic carcinoma cells to TBT induced a decrease in AMP-activated protein kinase (AMPK), leading to the impairment of glucose metabolism and uptake [15]. TBT and TPT derivatives induced apoptosis by decreasing BCL-2 expression and increasing BAX expression, but they exerted a negligible effect on P-gp efflux activity [16]. In addition, the conjugation of organotin compounds with compounds of different origins has been attempted to increase their biological activity, showing potent cytotoxic activity associated with the inhibition of DNA synthesis, apoptosis, and autophagy induction [17,18]. Similarly, the synthesis of novel TPT(IV) compounds conjugated with oxaprozin or propionic acid derivatives were shown to be cytotoxic versus the PC-3, HEP2G, HT-29, and MCF-7 cell lines, very likely owing to the induction of NO/ROS production and autophagosome formation following the high rate of tin uptake in comparison to platinum [19]. Conjugation with a carbohydrate-based scaffold or natural compounds was shown to improve organotin compounds’ solubility and potential anticancer activities. Di- and triorganotin compounds conjugated to D-(+)-galacturonic acid were reported to be cytotoxic versus MCF-7 and HCT-116 at submicromolar concentrations by affecting the mitochondrial transmembrane potential (Δψm) [20]. TBT(IV) ferulate (TBT-F), resulting from the conjugation of organotin with a ferulic acid natural compound with antioxidant properties, acquired pro-oxidant properties once conjugated with TBT(IV). This led to an increase in the biological activity of TBT-F by inducing autophagy through ER stress caused by ROS production in colon cancer cells [21,22]. Further studies have elucidated the effects of TBT or triphenyl tin (TPT) in vivo. A TPT carboxylate derivative counteracted the tumorigenesis of prostate cancer cells (PCa) in vivo in transgenic mice through the inhibition of AKT signaling [23], without notable toxic effects. 

Thus, despite most authors focusing on the induction of the apoptotic form of RCD as the main mechanism possibly involved in the potential anticancer effect of organotin compounds [24,25,26], the range of molecular targets presumably involved is highly heterogeneous and awaits further studies and clarifications. Moreover, given the multiple potential activities of organotin derivatives towards cancer cells, the relationships among structures, physiochemical properties, and biological activities of this class of compounds, and whether a difference exists in the effect they exert on nontumorigenic cells compared with tumorigenic cells are still elusive. 

In the present study, we explored the physiochemical properties of TBT-O and TBT-Cl derivatives in a hydrophilic environment and their dose-dependent cytotoxic effects towards representative high-tumorigenic and nontumorigenic cells in comparison with a tributyltin trifluoroacetate (TBT-OCOCF3). This organotin compound was specially designed and synthetized to find a possible balance between toxicity and feasible antitumor activity. The possible mechanisms involved in the effects exerted by the compound on cell death were also investigated. Finally, based on the obtained results, a strategy of a combination treatment specifically aimed at enhancing the potential anticancer activity of similar organotin compounds is proposed. 

## 2. Results

### 2.1. NMR Characterization of Structure, Solubility, and Aggregation

The structures of the three investigated TBT compounds, TBT-O, TBT-Cl, and TBT-OCOCF_3_, were confirmed by NMR spectroscopy using ^1^H-NMR, ^1^H-^1^H COSY, and ^1^H-^13^C HSQC experiments (Figure S1). The chemical shifts obtained in DMSO-*d_6_* and phosphate buffer at pH 7.4 are listed in Table 1. The chemical shift observed for the hydrogens in all compounds shows an accentuated shielding effect by Sn, which causes the signal of the CH2 group directly attached to it to occur at higher fields than those of the other two CH2 groups. The effect extends both to the hydrogens and the carbon of the CH2-1 group. This particular effect has already been observed in other cases [27]. 

The solubility was determined by preparing a 600 µL solution for each compound in the DMSO and buffer solution with a nominal concentration of 400 µM. The effective concentrations were measured at 37 °C using 3-(trimethylsilyl) propionic 2,2,3,3-*d_4_* acid (TSP) as an internal standard. The experimental and nominal values were almost identical for the DMSO solutions, whereas the measured values were significantly lower in the buffer solution. The three compounds showed solubility in the range of 40–70 µM in phosphate buffer, except for TBT-O, which was slightly less soluble (Table 2).

The aggregation state of the three compounds in the aqueous buffer was determined by measuring their hydrodynamic radii ($R_{h}$) using 1,4-dioxane as an internal standard [28]. The hydrodynamic radius of each compound ($R_{h,comp}$) can be calculated using Equation (1):

(1) $$R_{h,comp}=R_{h,dioxane}\frac{D_{dioxane}}{D_{comp}}$$

where $R_{h,dioxane}$ is the hydrodynamic radius of dioxane (2.12 Å). The diffusion coefficients of dioxane ($D_{dioxane}$) and the compound ($D_{comp}$) were measured using pulse-field gradient experiments. The results in DMSO show that all three compounds have a similar molecular volume (Table 2), even though a higher value of $R_{h}$ should be expected for TBT-O. Based on the $R_{h}$ value, we can conclude that, in DMSO, it is present as a monomer of its hydrated form, i.e., tributyltin hydroxide (TBT-OH), as shown in Table 1.

When compared with those obtained in DMSO, the $R_{h}$ values in phosphate buffer were higher for all compounds, except TBT-OCOCF_3_ (Table 2). This latter result can be explained by a hydrophobic collapse event caused by water, but it also indicates that this compound does not aggregate in the buffer solution. On the other hand, TBT-Cl shows the highest $R_{h}$ value in DMSO, implying that this compound is highly aggregated at its maximum concentration in the buffer solution. We performed a series of ^1^H-NMR spectra experiments, lowering the TBT-Cl concentration to 25 µM without noticing a difference in the line shape (data not shown). This observation indicates that this compound also remains aggregated at lower concentrations.

TBT-O also shows an increase in its $R_{h}$ from DMSO to phosphate buffer. Two effects can contribute to increasing this value: presence of the oxide or formation of aggregates. We noticed a chemical shift difference between DMSO and phosphate buffer for hydrogens in positions 1 and 2, which might be compatible with the presence of the oxide TBT-O (Table 1).

**Figure 1:**
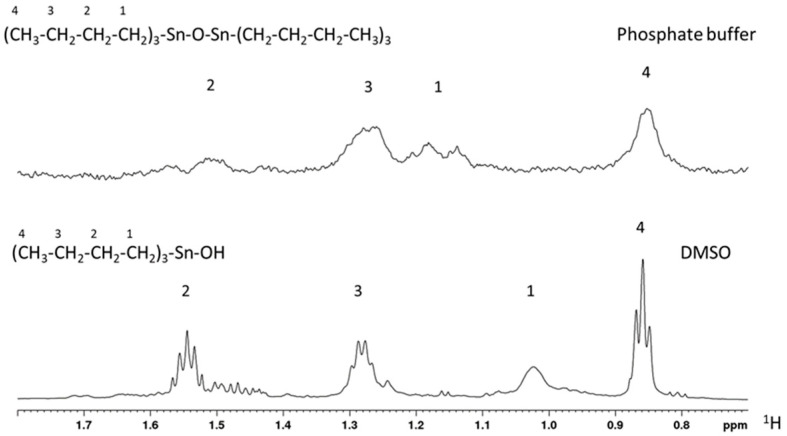
^1^H-NMR spectra for TBT-O in its dimeric (**upper**) and monomeric (**bottom**) forms acquired in phosphate buffer and DMSO, respectively.

However, we also observed a significant broadening for all signals in the water environment (Figure 1), which are larger than expected for converting the monomer into the dimer. For this reason and for the significant increment in $R_{h}$, we concluded that TBT-O is aggregated in phosphate buffer at 42 µM. 

Finally, all compounds were stable in DMSO and phosphate buffer for several days, as judged from the invariance of the corresponding ^1^H-NMR spectra (data not shown).

### 2.2. Effect of Organotin Compounds on the Metabolic Activity of CAL27, MCF-10A, U937 Cells, and PBMCs

Firstly, *an MTT assay was performed* as a biological indicator of the cellular metabolic activity, proliferation, and cytotoxicity in high-tumorigenic CAL27 cells and in nontumorigenic MCF-10A cells exposed to the three organotin compounds under investigation (TBT-O, TBT-Cl, and TBT-OCOCF_3_) and to the metal-derived anticancer drug, cisplatin, as a reference compound. In parallel, peripheral blood mononuclear cells (PBMCs) of healthy individuals and U937 cells were used as cellular models suitable for a cytotoxicity investigation. Five thousand cells were treated with the compounds at concentrations ranging from 0.625 µM to 80 µM, and, after 24 h, the formazan production was assessed. The results, expressed as half-maximal inhibitory concentration, are shown in Table 3. Regarding the high-tumorigenic CAL-27 cells, the results show that TBT-OCOCF_3_ exhibited an IC_50_ of 2.45 ± 0.14, i.e., lower than TBT-O (13.18 ± 3.70) but higher than TBT-Cl (0.91 ± 0.53). On the other side, CAL-27 cells were much more resistant to cisplatin (138.60 ± 59.52) than all of the tested organotins. Regarding the nontumorigenic MCF-10A cells, PBMCs, and U937 cells, Table 3 shows that, even in these cells, TBT-OCOCF_3_ is more inhibitory than cisplatin. TBT-O showed a metabolic inhibitory activity similar to that of TBT-OCOCF_3_ in PBMCs and U937 cells, respectively, while it exerted less inhibitory activity against MCF-10A cells (4.93 ± 2.33). Conversely, TBT-Cl *is* the most potent inhibitory compound showing an IC_50_ of approximately or lower than 1 μM in all tested cells. 

### 2.3. Effects of Triorganotin Compounds on Cell Viabilities of CAL-27 and MCF-10A Cells

**Figure 2:**
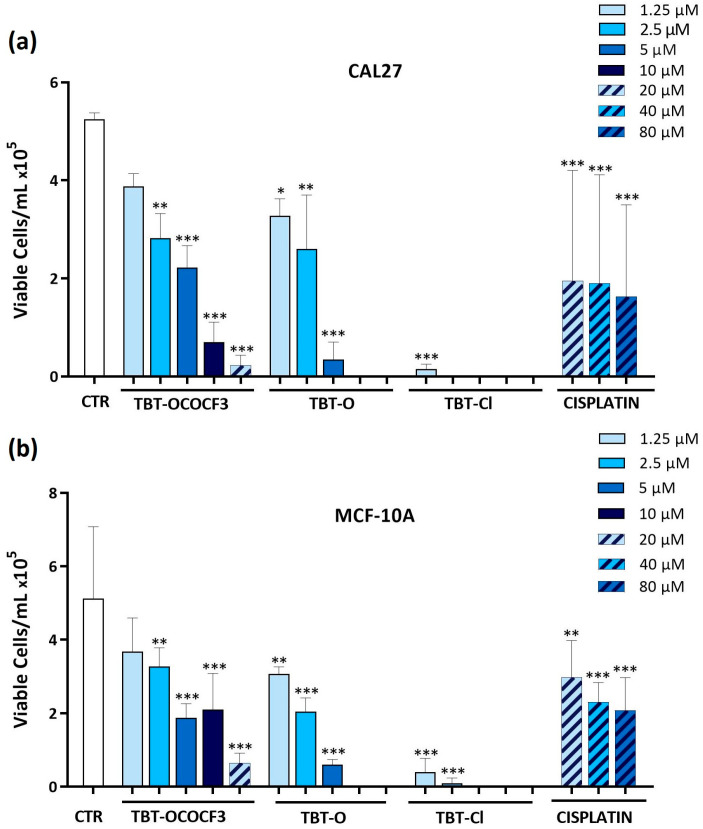
Absolute number of viable (**a**) CAL-27 and (**b**) MCF-10A cells, evaluated as negative by staining with the trypan blue exclusion test, following 24 h of treatment with the triorganotin compounds or cisplatin at concentrations ranging from 1.25 to 80 μM. * *p* ≤ 0.03, ** *p* ≤ 0.002, and *** *p* < 0.001 versus the control group. Data are expressed as the mean + SD and refer to three experiments performed in triplicate.

The viabilities of the CAL-27 and MCF-10A cells were determined by the trypan blue exclusion test after the treatment of these cells (1 × 10^5^) with TBT-O, TBT-Cl, and TBT-OCOCF_3_ at concentrations of 1.25 to 20 μM. As a reference compound, cisplatin was used only at higher concentrations of 20–80 μM, owing to the intrinsic resistance of the cell lines observed in the MTT assay and preliminary trypan blue exclusion tests. The absolute number of cells showing plasma membrane integrity that were recovered from each test sample was assessed after 24 h of culture. Regarding the high-tumorigenic CAL-27 cell line, TBT-O exerted dose-dependent cytotoxic effects, causing the complete inhibition of trypan-blue-negative cells recovered at 10 µM and 20 µM (Figure 2a). Furthermore, TBT-Cl greatly decreased the number of viable cells at all of the concentrations tested. Even TBT-OCOCF_3_ inhibited the CAL-27 cells’ viability in a dose-dependent manner but showed poor cytotoxicity at 1.25–5 µM and higher cytotoxicity at 10 µM and 20 µM (Figure 2a). With regard to MCF-10A cells, TBT-O caused dose-dependent cytotoxic effects, with the viability inhibited by 90% at 5 µM and by 100% at 10 µM and 20 µM (Figure 2b). TBT-Cl equally inhibited cell viability by approximately 90% or more at all of the tested concentrations (Figure 2b).

The addition of TBT-OCOCF_3_ more slightly affected the decrease in MCF-10A viable cells, with an inhibition of viability by 90% only at 20 µM (Figure 2b). Taken together, these data suggest a milder cytotoxic activity of TBT-OCOCF_3_ in comparison with the other triorganotin derivatives against the low-tumorigenic cells. Interestingly, the CAL-27 and MCF-10A cells were resistant to cisplatin treatment even at the higher concentration tested (Figure 2a,b). When the same assay was performed with the less cytotoxic TBT-OCOCF_3_ organotin compound in nonadherent U937 cells, after 24 h of treatment at 10 µM, only less than 10% of the viable cells in comparison with the control samples was still detectable (Figure S2 in the Supplementary Materials). This result is coherent with that observed for the MTT assays in the same cells (Table 3). 

The overall results of these experiments show an evident and, in some cases, dramatic dose-dependent disappearance of trypan-blue-negative cells showing plasma membrane integrity after TBT treatment. Surprisingly, however, this disappearance was not mirrored by a corresponding increase in the number of trypan-blue-positive dead cells. The presence of still morphologically intact, trypan-blue-positive cells was detectable, even if in small amounts, only in samples treated with the higher concentrations tested (10 µM and 20 µM), where, in any case, the sum of trypan blue-negative plus trypan blue-positive cells was never equivalent to the absolute number of control cells (data not shown). 

### 2.4. Interaction of TBT-OCOCF_3_ with 12 bp-DNA

**Figure 3:**
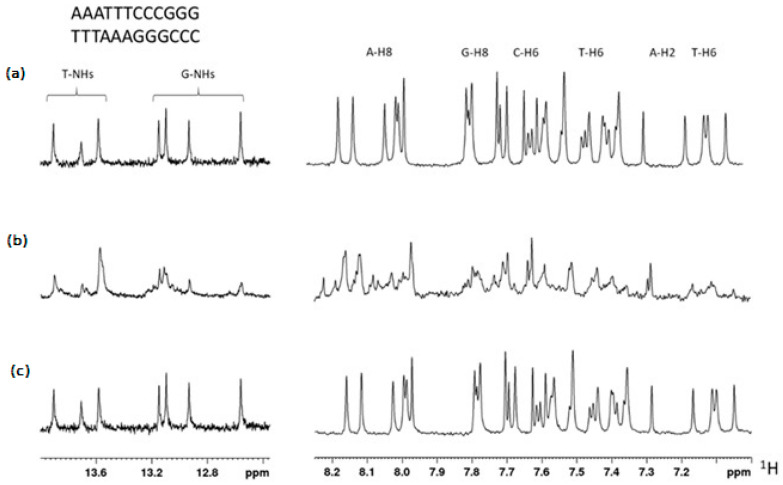
(**a**) ^1^H-NMR spectrum region of the T- and G-NH groups (left) and aromatic signals of the 12 bp DNA duplex, the sequence of which is indicated at the top. Signal types were assigned by a combination of chemical shift values and an ^1^H-^1^H TOCSY experiment. (**b**) Spectrum obtained after 6 h of incubation with two equivalents of cisplatin. (**c**) Spectrum obtained after 6 h of incubation with five equivalents of TBT-OCOCF_3_.

In order to investigate the mechanisms underlying the cytotoxicity exerted by TBT-OCOF_3_, a first approach was to verify whether they were based on a direct interaction with DNA, similar to what was described for metal derivatives such as cisplatin. To this purpose, we investigated if TBT-OCOCF_3_ could bind to DNA using NMR. For TBT-OCOCF_3_, the 1D and 2D spectra of 12 bp DNA were acquired in the presence or absence of the compound. As a positive control for the interaction, cisplatin was assayed, which forms adducts or inter- and intra-strand crosslinks resulting in interference in the replication machinery, G2/M cell arrest, and cell death by apoptosis or necrosis [29]. A solution containing 0.2 mM of the duplex, whose sequences are indicated in Figure 3, was annealed directly in the NMR tube. The double-helix formation was confirmed by the appearance of low-field signals belonging to the T- and G-NH groups (Figure 3a, left panel). The DNA’s aromatic signals were assigned to the different types of ^1^H nuclei using a combination of ^1^H-NMR and ^1^H-^1^H TOCSY experiments (Figure S3 in the Supplementary Materials) and chemical shift values (Figure 3a, right panel).

After incubation with two equivalents of cisplatin for six hours, the NMR spectrum showed evidence of the interaction (Figure 3b). On the contrary, incubation with two or five equivalents of TBT-OCOCF_3_ did not result in any observable change in the NMR spectrum (Figure 3c). The signals of the compound were clearly observable in the mixture, indicating that it was available in solution for the interaction. These results indicate that TBT-OCOCF_3_ does not interact with the 12 bp DNA, suggesting that it is very likely that, differently from cisplatin, a direct interaction with DNA is not the mechanism of action of the cytotoxicity exerted by this compound or possibly other compounds of this class.

### 2.5. Inhibition of Glucose Uptake by Triorganotins

**Figure 4:**
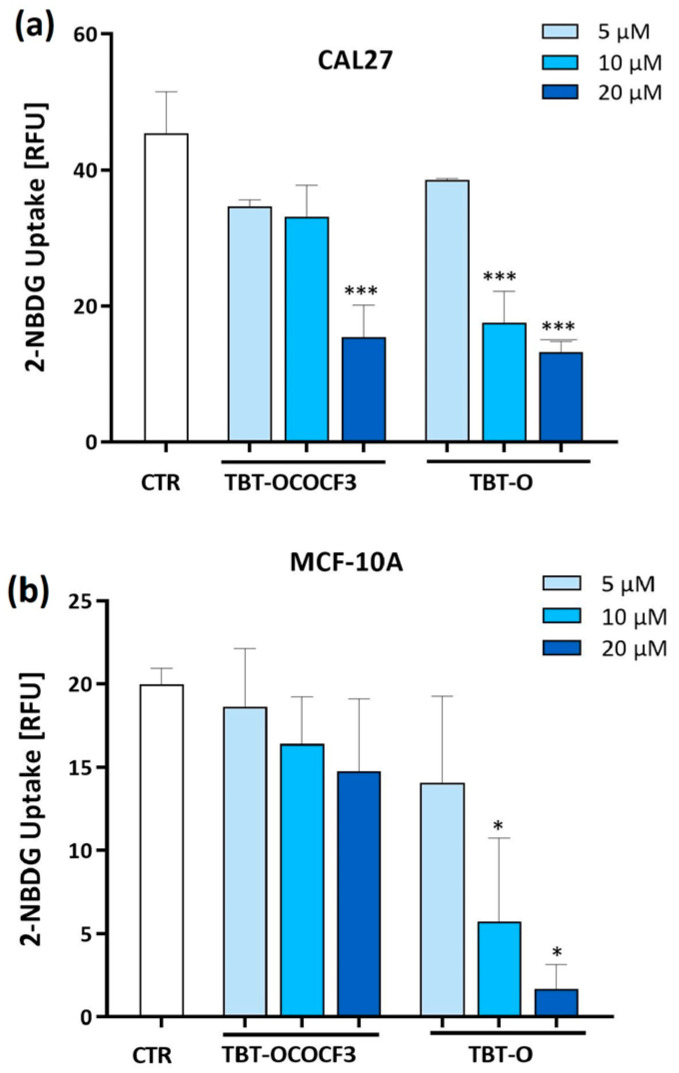
Glucose uptake assessed by flow cytometry analysis using a deoxy-glucose analog 2-NBDG as a fluorescent indicator in (**a**) CAL-27 cells and (**b**) MCF-10A cells treated with the organotin derivatives for 1 h. Data are the means + SD from two experiments in duplicate. * *p* < 0.03 vs. control; and *** *p* < 0.001 vs. control.

Glucose metabolism plays a key role in tumor cell growth. Therefore, it was investigated whether the cytotoxicity exerted by the triorganotin compounds could be related to the inhibition of glucose uptake in the tumor or in the nontumorigenic cell lines. CAL-27 and MCF-10A cells were treated with TBT-O and TBT-OCOCF_3_, the organotin compound that exhibited the lower toxicity. The cells were treated at 5 µM, 10 µM, and 20 µM in the presence of a fluorescent analog of glucose-2 NBDG for 1 h. The results show that TBT-O at 10 μM and 20 μM significantly inhibited glucose uptake in the CAL-27 cells (Figure 4a) and MCF-10A cells (Figure 4b). In contrast, TBT-OCOCF_3_ induced an immediate and highly significant inhibition of glucose uptake in the CAL-27 cells at 20 µM (Figure 4a), while it did not induce a similar inhibition in the MCF-10A cells (Figure 4b).

Collectively, these data show that the organotin-derived compounds induce an immediate glucose uptake inhibition related to their cytotoxicity.

### 2.6. Effects of TBT-OCOCF_3_ on Cell Death in CAL-27 and MCF-10A Cells

Data on the inhibition of the metabolic activity and the viable/dead cell count indicate that the CAL-27 cells were resistant to the cytotoxic effects exerted by cisplatin but quite susceptible to those induced by organotin derivatives, including TBT-OCOCF_3_. However, except for a certain relationship between toxicity and glucose uptake inhibition, the mechanisms involved in the cytotoxic effects of the organotin derivatives, particularly TBT-OCOCF_3_, were still hidden. To better understand the disappearance of viable cells in the TBT-treated samples, we investigated the effects of TBT-OCOCF_3_, the compound that exhibited the milder cytotoxic activity, on the death of CAL-27 and MCF-10A cells using an annexin-V (Anx)/7-AAD double-staining assay. This assay allows us to simultaneously recognize viable cells (Anx-/7-AAD-), presumably early apoptotic cells (Anx+/7-AAD-), late apoptotic/secondary necrotic cells (Anx+/7-AAD+), and primary necrotic/lacking plasma-membrane-integrity cells (Anx-/7-AAD+). The cells were treated with 20 µM TBT-OCOCF_3_ (i.e., at a concentration showing appreciable toxicity), and, after 4 h (i.e., at a time when most of the cells were still detectable), the Anx+ and 7-AAD+ positivity was assessed using flow cytometer analysis. Moreover, to further investigate the mechanisms involved in TBT-OCOF_3_-induced cell death, attention was focused on emerging evidence that a complex crosstalk between apoptotic RCD and autophagy could play an essential role in influencing the survival/death of malignant cells and, consequently, the success of the chemotherapeutic treatment of cancer [30]. Therefore, tumorigenic CAL-27 cells were also subjected to cotreatment with the autophagy inhibitor wortmannin at 0.5 µM, in addition to TBT-OCOF_3_. Table 4 shows that TBT-OCOCF_3_ induced approximately 4% Anx+/7-AAD- and 8% Anx-/7-AAD+ cells, compared with approximately 11% and 0.6%, respectively, in the control cells. A low but detectable amount of Anx+/7-AAD+ double-positive cells were induced by the treatment of TBT-OCOCF_3_, while this subset, as well as the Anx-/7-AAD+ subset, was practically absent in the cells treated with diluent alone as a control. Regarding MCF-10A cells, Table 4 shows that, after TBT-OCOCF_3_ treatment for 4 h, the percentage of Anx+/7-AAD- cells significantly increased to 24.68% compared to 3.68% in the control cells, while that of the Anx-/7-AAD- cells significantly decreased from 75.96 to 14.16 compared to the control cells. Nevertheless, Anx+/7-AAD+ double-positive cells significantly increased at a higher percentage of 47.59 after treatment with respect to the 9.36% in the control cells. Therefore, TBT-OCOCF_3_ treatment after 4 h induced both single Anx+ and, at the same time, double Anx+/7-AAD+ cells. Conversely, the percentage of Anx-/7-AAD+ was similar in the treated and control cells. This is coherent with the significant increase in the dead cell count by the trypan blue assay following 24 h of TBT-OCOCF_3_ treatment, as shown in Figure 2b.

Regarding the effects of the autophagy inhibitor wortmannin on TBT-OCOCF_3_-induced cell death, Table 4 shows that the cotreatment did not significantly modify the percentage of Anx+/7-AAD-, while it increased the percentage of Anx+/7-AAD+ cells significantly, and that of the Anx-/7-AAD+ cells highly significantly, to approximately 6% and 29%, respectively. In addition, a cell cycle analysis carried out in the MCF-10A cells by PI staining revealed that a significant percentage of the TBT-OCOCF_3_-treated cells shifted from phase G2 (24%) to phase sub-G1 at 24 h after treatment, showing a significant increase in hypodiploid nuclei (15.4%), which are a typical feature of apoptotic cells, in comparison with the control cells (Figure S4 in the Supplementary Materials).

These data suggest that TBT-OCOCF_3_-induced cell death resembling early and/or late apoptotic RCD, which might mainly be detectable in MCF-10 cells, after 4 h of treatment at the toxic concentration. However, at the same time, TBT-OCOCF_3_ also induced a proneness to plasma membrane disruption, a typical feature of primary or secondary necrosis and other forms of RCD, both in the tumorigenic and the nontumorigenic cells used, as shown by the 7AAD+ positivity. In particular, this proneness was more pronounced in the MCF-10 cells in comparison with CAL-27 cells. Moreover, data on the effects of wortmannin were suggestive of a possible role of autophagy as a regulator of TBT-OCOCF_3_-induced cell death.

### 2.7. Effect of TBT-OCOCF_3_ on Cell Death and Autophagy in U937 Cells

**Figure 5:**
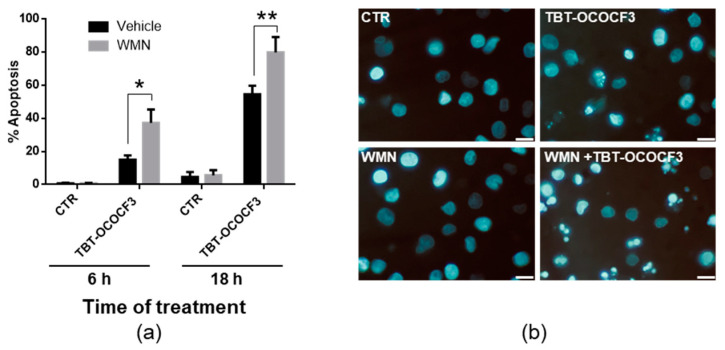
Fluorescence microscopic analysis of U937 cells treated with TBT-OCOCF_3_ (1 µM) alone or with wortmannin (WMN, 0.5 µM) after staining with the fluorescent DNA-binding dye Hoechst. (**a**) The % of cells showing features of apoptosis after 6 and 18 h of treatment from 6 frames randomly selected from three independent experiments (2 frames each; mean + SD). * *p* < 0.01; and ** *p* < 0.001. (**b**) Representative images of cells subjected to different experimental conditions for 18 h are displayed. In the samples cotreated with WMN plus TBT-OCOCF_3_, cells showing typical characteristics of advanced apoptosis with nuclei present as one or more groups of featureless, bright, and spherical beads can be abundantly detected. Objective 63×, scale bar = 10 μm.

Based on the results of the cotreatment with TBT-OCOCF3 and wortmannin in CAL-27 cells, we decided to further investigate whether a crosstalk between apoptotic RCD and autophagy was effective in controlling cytotoxicity induced by organotins. To this purpose, nonadherent U937 cells, which are highly susceptible to TBT-induced cytotoxicity (Table 3; Figure S2 in the Supplementary Materials), were used. The cells were treated with 1 μM TBT-OCOCF_3_ alone or with 0.5 μM wortmannin for 6 and 18 h. The percentages of cells showing morphological features of apoptotic cells were then evaluated by a microscopic analysis following staining with the fluorescent DNA-binding dye Hoechst. The choice for time and TBT-OCOCF_3_ concentration was based on the previous results and those of preliminary experiments, showing that, in the selected experimental conditions but not at higher concentrations of TBT-OCOCF_3_, appreciable amounts of intact cells were still recoverable. In this round of experiments, fluorescence microscopic analysis indicated that, after 6 h, 15% of the U937 cells treated with TBT-OCOCF_3_ alone showed morphological characteristics of apoptosis, while the addition of wortmannin significantly increased the portion of cells positive for apoptotic signs, overall up to nearly 37% (Figure 5a). A similar trend was observed when the timing was prolonged to 18 h but with much higher percentages of apoptotic cells (Figure 5a). As shown by representative images, the nuclear morphology of the U937 cells detected by Hoechst labeling revealed that the cotreatment of wortmannin even with a low dose of TBT-OCOCF_3_ determined a considerable induction of cells with apoptotic features (Figure 5b).

**Figure 6:**
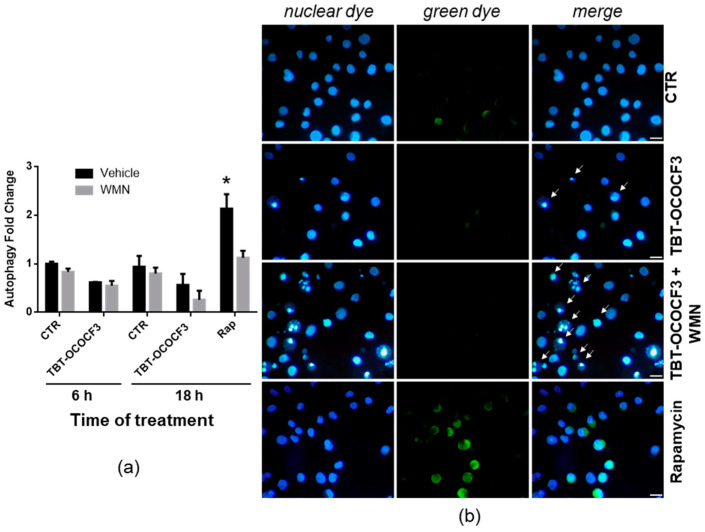
Detection of the autophagic flux in U937 cells treated with TBT-OCOCF_3_ (1 µM) alone or with wortmannin (WMN, 0.5 µM) after double staining with a fluorescent green dye that selectively labels autophagic vacuoles and with the fluorescent DNA-binding dye DAPI (Autophagy Assay kit, Abcam). As a positive control for autophagy, cells were treated with rapamycin 0.5 µM (Rap) for 18 h. (**a**) Autophagy fold change with respect to the control cells measured by green fluorescence emission using a fluorescence microplate reader after 6 and 18 h of treatment. The results are from three independent experiments performed in duplicate (mean + SD). * *p* < 0.01 vs. all groups. (**b**) Representative images of cells subjected to different experimental conditions after 6 h of treatment. In the samples treated with TBT-OCOCF_3_, typical features of apoptosis, especially evident following cotreatment with WMN, can be detected (white arrows). Fluorescent green autophagic vacuoles can be clearly detected only in the cells treated with rapamycin or, occasionally, in the control cells. Objective 40×, scale bar = 10 μm.

To obtain direct information on the role of autophagy in regulating cytotoxicity induced by TBT-OCOCF_3_, the autophagic flux in viable cells was then assessed using a dye that selectively labels autophagic vacuoles. U937 cells were treated with 1 μM TBT-OCOCF_3_ alone and with the same amount of TBT-OCOCF_3_ plus 0.5 μM wortmannin for 6 and 18 h. These experimental conditions were the same, shown in Figure 5, in which increased levels of apoptosis following cotreatment with wortmannin were clearly detectable. As a positive control of autophagy induction, cells were treated for 18 h with 0.5 μM rapamycin alone or with the addition of the inhibitor wortmannin. All samples were analyzed for fluorescence emission using a fluorescence microplate reader at the indicated times. As illustrated in Figure 6a, only the rapamycin treatment determined a significant increase in autophagic-specific fluorescent green signals compared to the control cells. As expected, cotreatment with wortmannin significantly inhibited rapamycin-induced autophagy. Moreover, no significant change in fluorescence signals was found in the cells treated with TBT-OCOCF_3_ as a single treatment or in combination with wortmannin after 6 or 18 h of treatment. Similar results were obtained when aliquots of the samples were analyzed by fluorescence microscopy. As shown in Figure 6b, autophagy, evidenced by green fluorescence, was present only in U937 cells treated with rapamycin.

Moreover, also in this set of experiments, the nuclear morphology of the U937 cells was detected by double staining with DAPI fluorescent dye. Again, the results exhibited that a cotreatment of TBT-OCOCF_3_ and wortmannin, at the concentration utilized to detect autophagy, determined a clear increase in cells with apoptotic features with respect to the samples treated with the TBT alone (Figure 6b, white arrows). These results confirmed what was previously observed using Hoechst as a DNA-binding dye to detect apoptosis, as shown in Figure 5.

These findings highlighted that wortmannin was able to noticeably increase the level of apoptotic RCD induced by a low dose of TBT-OCOCF_3_ early after treatment. However, this was not owing to the inhibition of an autophagic flux that was not observed in cells treated with the compound in the same experimental conditions.

## 3. Discussion

The present in vitro comparative study shows that tributyltin derivatives were able to differently but always strongly affect cell proliferation, cell viability, and cell death in a tumorigenic, as well as in a nontumorigenic, cell line.

The biological activity of organotin(IV) compounds should depend on different features, such as the nature of the organic moiety and donor ligands attached to the tin atoms. Therefore, in this study, the major chemical physical parameters of the studied compounds were first ascertained. In particular, the solubility of the compounds in hydrophilic environments was checked through NMR before analyzing their biological effects. The NMR data show that a broad dose–effect range lower than the threshold of the solubility in phosphate buffer could be effectively utilized in biological assays with the tributyltin compounds. In any case, TBT compounds were dissolved in DMSO to make stock solutions for storage at concentrations high enough to warrant dilutions of at least 1/1000 with phosphate buffer before their use for in vitro and in vivo assays. Moreover, the results indicate that, in general, except for TBT-Cl, both TBT-OCOCF_3_ and TBT-O did not remain aggregated at the concentrations utilized for the biological assays.

Regarding the biological activity, to obtain information on the possible anticancer potential of the tributyltin derivatives, as a first approach, the effects of the compounds on the cellular metabolic activity and on the cell viability of a tumorigenic and a nontumorigenic cell line were investigated. The effects towards PBMCs from healthy individuals and towards the nonadherent U937 cell line were also investigated for comparison. The results of this part of the study undoubtedly reveal the high cytotoxic properties of the tributyltin compounds. Among the three tested tributyltins, TBT-Cl was found to be significantly and equally highly inhibitory for the metabolic activity (IC_50_ of approximately 1 µM) and cytotoxic both at high and low concentrations versus all tested cell lines. This might be owing to the electronegative characteristic of the chloride ion, which enhances the reactivity of the metal by attracting the electron cloud. Conversely, TBT-OCOCF_3_ and TBT-O inhibited the metabolic activity similarly in all cell lines at higher values between 2 µM and 4 µM. In particular, TBT-OCOCF_3_ (i.e., the synthesized tributyltin whose chemical and biological features were defined for the first time in this study) reduced the number of intact cells recovered after treatment in a dose–effect fashion and, to a lesser extent, in the MCF-10A cells with respect to the CAL-27 cells. Nevertheless, all cell lines assayed were more resistant to cisplatin treatment in comparison with the tributyltin derivatives. Although these results provide a clear picture of the high cytotoxic potential of tributyltins, they did not, however, provide sufficient information on events triggered by the compounds in the cells underpinning this cytotoxic activity. The substantial decrease in the metabolic activity, as assessed by the MTT assay, corresponds to an evident decrease in the number of cells in an active phase of their life. Nevertheless, this could be due to a metabolic block, to block in their cell cycle, to the entering of a high number of cells on the road towards one of the forms of RCD, or simply to the disappearance of intact cells in the treated samples due to the fact of their bursting because of primary or secondary necrosis. Even the trypan blue assays, despite demonstrating that the lowering of the metabolic activity was strictly associated with the lowering of the number of cells showing plasma membrane integrity, were helpful in elucidating this aspect. Unexpectedly, in our assays, the decrease in trypan-blue-negative, intact, and presumably viable cells was not balanced by a concomitant corresponding increase in trypan-blue-positive, intact, and presumably dead cells. On the other hand, viable trypan-blue-negative cells could not be distinguished from early apoptotic cells that they too were trypan blue negative, nor were the cells undergoing forms of RCD different from apoptosis with a loss of plasma membrane integrity distinguishable from primary or secondary necrotic cells, which were all trypan-blue-positive.

Thus, based on the results of the first part of the study, the successive phases mainly focused on the novel TBT-OCOCF_3_ derivative, on its potentiality as a prototype anticancer candidate, on the attempt to characterize its cytotoxicity precisely, and, more specifically, on the type of induced cell death. However, a complex scenario was also found as a result of this part of the study. Experiments performed in the tumorigenic and nontumorigenic cells under study showed that a quite evident percentage of dead cells following TBT-OCOCF_3_ treatment could be detectable only at higher concentrations of approximately 20 µM. In these experimental conditions, however, only a very low amount of total still-intact cells could be recovered after 24 h from the treated samples. Nevertheless, to understand which form of cell death could be induced by TBT-OCOCF_3_ at the concentration mentioned above, cells with features of early and late apoptotic RCD and of necrotic death were distinguished by flow cytometry analysis following annexin-V/7AAD double staining early after treatment. The results show that a noticeable percentage of the MCF-10A cells that were recovered after 4 h of treatment with TBT-OCOCF_3_ at 20 µM had the characteristics of early apoptosis. Nevertheless, a high percentage of cells were positive for both annexin-V and 7-AAD or for 7-AAD alone, indicating a coexistence of cells resembling early apoptotic, late apoptotic, and primary and secondary necrotic cells. Interestingly, the complexity of cell death induced by TBT was observed in a previous study demonstrating that at least two independent pathways were implicated in caspase-3-independent neuronal cell death caused by this compound [31].

Even though our results add an expected complexity to the phenomena under investigation (i.e., the actual fate of cells exposed to the selected tributyltin), they helped to address our efforts towards understanding, at least in part, the mechanisms that could control this network of cell-death-regulating signaling. Some key points concerning this aspect were defined. For example, our results demonstrate that cytotoxicity induced by the assayed triorganotin derivatives was found not to be owing to DNA binding. Indeed, unlike cisplatin, TBT-OCOCF_3_ does not bind DNA, as demonstrated by the NMR analysis. This finding opens the way for developing TBT-OCOCF_3_-based compounds as potential anticancer agents in cisplatin-resistant tumors. Moreover, the cell cycle analysis showed that the cytotoxicity induced by TBT-OCOCF_3_ was associated with a block in the G0/G1 and G2 phases and to the entry in the sub-G1 phase for the few intact cells that could be recovered after the treatment. Conversely, a very low percentage of cells were in the S phase. Similar findings were reported by other authors, who demonstrated that halogenated tin phosphinoyldithioformate complex-derived compounds induced the inhibition of proliferation but not accumulation in the S phase of the cell cycle [32]. Furthermore, the anticancer activity of di- and triorganotin(IV) compounds associated with D-(+)-galacturonic acid was reported to be associated with a block in the G0/G1 phase [20]. Therefore, our data and those reported by other authors lead us to conclude that even cells that can overcome the cytotoxicity after exposure to organotin compounds cannot proliferate at all, thus explaining the extremely low number of cells recovered by us following treatment and, most importantly, supporting their possible development as anticancer agents.

Another point of our study is the finding of a certain relationship between the cytotoxicity and the inhibition of the glucose metabolism of tin derivatives. We showed that TBT-OCOCF_3_ is more effective in inhibiting glucose uptake in tumorigenic than in nontumorigenic cell lines. Glucose is a primary font of energy, and a hypothetical association between glucose metabolism and the effects of triorganotin derivatives was demonstrated in the human pluripotent embryonic carcinoma cell line NT2/D1 in which exposure to tributyltin inhibited glucose-6-phosphate and fructose-6-phosphate production via the inhibition of the transporter GLUT-1 [15]. In our model, CAL-27 exhibited a higher expression of membrane GLUT-1 with respect to MCF-10A cells (data not shown). Given that the dysregulation of the energy metabolism is a fundamental hallmark of cancer cells [33] and that glycolysis-related cancer cell survival is mediated by glucose transporters upregulated in some types of cancer [34], our results could suggest that compounds similar to TBT-OCOCF_3_ could selectively act as cell-death inducers in tumors but not in normal cells. Nevertheless, it should be noted that TBT-OCOCF_3_ markedly decreased the number of viable cells at concentrations that did not significantly affect glucose uptake. Thus, the role of the inhibition of glucose metabolism in the cytotoxicity exerted by tin compounds has yet to be elucidated.

The other key point addressed in our study is that a crosstalk between autophagy and cell death could be one intricate cell regulatory process triggered by tributyltins. The role of autophagy in controlling the fate of cancer cells is still a debated topic [30,35]. Autophagy is a double-edged sword whose activation/inhibition could favor tumor exhaustion [36]. Due to the evidence indicating a strict interaction between cell signaling controlling autophagy and different forms of cell death, including RCD and necrosis [37,38,39], targeting autophagy has been proposed as a new, possible strategy for anticancer therapy [40,41]. To better define events controlling tributyltin-induced cytotoxicity in CAL-27 cells—in fact, to address the possible regulatory role of autophagy—we utilized a cotreatment with an autophagy inhibitor, wortmannin. The results of our experiments in CAL-27 cells showed a low percentage of annexin-V+/7AAD- positive cells following treatment with TBT-OCOCF_3_ alone, as expected, but the cotreatment with wortmannin led to the detection of an increased number of cells showing features of dead cells, including approximately 29% of annexin-V-/7AAD+ cells. This indicates a proneness to undergoing various forms of cell death, particularly necrosis, in tumorigenic cells in which signaling driving the autophagic flux was inhibited. Of fundamental importance, in this respect, for the continuation of the study was the utilization of nonadherent U937 cells as an experimental model, being more versatile for such an investigation. Even in the U937 cells, cotreatment with wortmannin significantly increased the percentage of dead cells treated with TBT-OCOCF_3_ with respect to those treated with TBT-OCOCF_3_ alone. In this case, it was possible to define that the increase in the number of dead cells should mainly be ascribed to cells resembling apoptotic cells. Interestingly, a shift of U937 towards a functional phenotype more susceptible to cell death due the fact of wortmannin cotreatment was observed even with low concentrations of TBT-OCOCF_3_ and/or a short incubation time. However, the hypothesis that the levels of tributyltin-induced signaling driving towards apoptotic RCD should be negatively controlled by a concomitant triggering of autophagy was not confirmed by experiments finalized to prove this hypothesis directly. In the U937 cells, a low concentration of TBT-OCOCF_3_ plus wortmannin induced twice the percentage of cells showing apoptotic features with respect to TBT-OCOCF_3_ alone, but no sign of autophagic flux induction was observed as a consequence of tributyltin treatment. Wortmannin has been shown to inhibit autophagy through its well-known ability to suppress the class III phosphoinositide 3-kinase (PI3K), thus blocking the phosphorylation of phosphatidyl inositol (PI) that generates phosphatidylinositol 3-phosphate (PI3P), whose production is essential for the initiation of autophagy [42,43]. However, in our model, the inhibitory effect of wortmannin on autophagy seems not to be involved in the increase in the cytotoxic response. Thus, the effect of wortmannin in counteracting resistance to undergoing death in tumor cells might still be owing to its specific role of inhibiting the PI3K/AKT pathway. This pathway has been indicated as a central cellular mechanism for the phosphorylation of factors involved in the survival and migration of tumor cells [44]. In addition, the PI3/AKT kinase mammalian target of the rapamycin (mTOR) pathway is altered in HNSCC tumors, and agents targeting it are in clinical development to be used in combination treatment with chemotherapy [45]. Moreover, it has been recently reported that the conjugation of tributyltin with natural phenolic phytochemicals, such as ferulic acid (i.e., a ligand moiety absent within the TBT-OCOCF_3_ structure), induced an increase in LC3II and p62 autophagic proteins that preceded cell death in colon cancer cells but neither apoptotic nor necroptotic cell death [21]. Interestingly, tumorigenic CAL-27 cells seem to undergo cell death through necrosis rather than apoptotic RCD following tributyltin treatment. In any case, our study, although it does not explain the effect of wortmannin, clearly indicates that a pharmacological intervention acting on the PI3K/AKT signaling pathway could dramatically potentiate the cytotoxic potential of TBT. Importantly, such a combination treatment seems able to overcome the resistance of tumor cells to the induction of death by tributyltins and to drive the fate of the treated cells towards a more specific and effective route of RCD.

This study established for the first time that the mechanisms underlying the induction of cell death by tributyltin derivatives seem unexpectedly very multifaceted. Based on our results, the induction of RCD or necrotic-related processes by TBT seems to not be a univocal phenomenon but rather an occurrence greatly dependent on the target cells, on the specific scaffold of the structure of the chemical derivatives, and on the concomitant modulation of specific cellular signaling pathways. Future studies are necessary to elucidate further the exact mechanisms underlying the cytotoxic effect of triorganotin derivatives and to promote the development of novel tributyltin compounds as anticancer agents.

## 4. Materials and Methods

### 4.1. Synthesis of Tributyltin Trifluoroacetate, Chemicals, and Reagents

The synthesis of tributyltin trifluoroacetate (TBT-OCOCF_3_) was performed by the dropwise addition of TFA (0.04 mL, 0.5 mmol) to (Bu_3_Sn)_2_O (0.13 mL, 0.25 mmol). The reaction mixture was stirred at room temperature for 25 min and evaporated under reduced pressure to obtain a white solid. The compound was recrystallized from hexane and characterized by ^1^H NMR, ^13^C NMR, and IR. Stability tests in deuterated solvents, such as CDCl_3_, CD_3_OD, D_2_O, and d6-DMSO, at 37 °C were conducted by recording the ^1^H NMR spectra at regular intervals. In all cases, no change in the ^1^H NMR spectra was observed even after several weeks.

IR (neat): ν = 2959, 2926, 2859, 1653, 1445, 1192, 1150, and 727 cm^−1^.

^1^H NMR (400 MHz, CDCl_3_) δ 1.73–1.54 (m, 6H), 1.49–1.23 (m, 12H), 0.92 (t, J = 7.3 Hz, 9H); ^13^C NMR (100 MHz, CDCl_3_) δ 161.2 (q, J_19F/13C_ = 39.3 Hz), 115.1 (q, J_19F/13C_ = 288.1 Hz), 27.4 (td, J_119Sn/13C_ = 21.3 Hz), 26.9 (tdd, J_119Sn/13C_ = 65.2 Hz, J_117Sn/13C_ = 62.4 Hz), and 17.3 (tdd, J_119Sn/13C_ = 337.9 Hz, J_117Sn/13C_ = 332.9 Hz) ppm.

^1^H NMR (400 MHz, CD_3_OD) δ 1.83–1.47 (m, 6H), 1.47–1.13 (m, 12H), 0.92 (t, J = 7.3 Hz, 9H) ppm.

^1^H NMR (400 MHz, D_2_O) δ 1.60–1.35 (m, 6H), 1.31–1.00 (m, 12H), 0.88–0.65 (m, 9H) ppm.

^1^H NMR (400 MHz, d6-DMSO) δ 1.68–1.38 (m, 6H), 1.36–1.18 (m, 6H), 1.18–0.96 (m, 6H), 0.84 (t, J = 7.3 Hz, 9H) ppm.

TBT-O, TBT-Cl, and cisplatin were purchased from Sigma-Aldrich (San Diego, CA, USA). All tributyltin compounds were diluted in DMSO and stored at 1 M before their utilization for the biological assays. Wortmannin (Sigma-Aldrich) was diluted in DMSO and stored at 100 mM.

In all experiments carried out with the TBT compounds, the control cells were exposed, for the same amount of time as the treated cells, to control diluent alone corresponding to the higher concentration of the compounds assayed.

### 4.2. Cells

The high-tumorigenic human head and neck squamous cell carcinoma (HNSCC), adherent CAL-27 cells, and nonadherent U937 lymphoblastoid cells were maintained in RPMI 1640 medium supplemented with 10% fetal bovine serum (FBS), 50 U/mL streptomycin, 50 U/mL penicillin, and 2 mM glutamine (CM; Gibco-Invitrogen, Paisley, Scotland, United Kingdom) in a humidified incubator at 37 °C and 5% CO^2^. The nontumorigenic human mammary epithelial MCF-10A cells (ATCC, NIH, MD) were kept in DMEM-F12, (Lonza, Switzerland) supplemented with 5% horse serum (Invitrogen, Thermo-Scientific, CA, USA), 0.5 µg/mL hydrocortisone, 50 ng/mL cholera toxin, 0.01 mg/mL human insulin (Sigma-Aldrich), 50 U penicillin/streptomycin, and 2 µg/mL epidermal growth factor (EGF, Tebu-Bio, Le-Perray-en-Yvelines, France). The PBMCs were isolated from the buffy coat collected from healthy adult donor volunteers, who were seronegative for HIV and hepatitis B and C viruses, enrolled in the Polyclinic Hospital Tor Vergata Transfusion Center for blood donation for therapeutic purpose. The donors authorized the use of the remaining leukocytes for research purposes, signing a consent form. Anonymized buffy coats were diluted in phosphate buffered saline at pH 7 (PBS), and mononuclear cells were separated using a Ficoll–Hypaque density gradient (Cederlane, Hornby, Ontario, Canada) at a ratio of cells:gradient of 1:2. The cells were then centrifuged for 30 min at 1800 RPM and washed twice in RPMI 1640 medium (Gibco-Invitrogen). The PBMCs were stimulated with IL-2 at 10 U/mL (Proleukin, Chiron, Amsterdam, the Netherlands) before treatment with the compounds under study.

### 4.3. Metabolic Activity and Viability Assay

The inhibition of cell metabolic activity, revealed by the reduction of the oxidative burst, was performed through a colorimetric method based on the reduction of tetrazolium salt MTT (3-(4,5-dimethylthiazol-2-yl)-2,5-diphenyltetrazolium bromide, Sigma-Aldrich), with the formation of formazan crystals solubilized following the addition of SDS lysis buffer. The MTT reagent was diluted in sterile PBS (phosphate buffered saline) and stored at a concentration of 5 mg/mL, while 20 g of SDS was diluted in 100 mL solution composed of 50 mL bidistilled water and 50 mL dimethylformamide (Sigma-Aldrich). The assay was performed by seeding 5 × 10^3^ cells in 100 µL into a 96-well plate in the presence or absence of different concentrations of organotin compounds and the reference drug. After 18 h of incubation at 37 °C, 10 µL of MTT was added, and, after 6 h, 100 µL of SDS was added. After overnight incubation, the optical density was read by a spectrophotometer (Packard, Spectral Count Microplate Photometer) at the wavelength of 570 nanometers. The amount of formazan produced was directly proportional to the number of alive cells. The results are expressed as the drug concentration required to inhibit 50% of the metabolic activity ± standard deviation (IC_50_ ± SD). The concentrations of the compounds to be tested and the length of the incubation time for the MTT assay were chosen based on preliminary experiments showing IC_50_ values in the low micromolar range for TBT at 24 h and very small amounts of cells still detectable after longer incubation times. A viability assay was performed using the trypan blue exclusion test, with the concentrations of the compounds and timing selected on the basis of the MTT assay and preliminary trypan blue exclusion tests.

### 4.4. NMR Spectroscopy

For the NMR studies, the compounds were dissolved in DMSO-d6 and a phosphate buffer (50 mM, 5% D_2_O, pH = 7.4) containing, as an internal standard, 2,2,4,4-tetradeuterumtrimethylsilylpropionic acid (TSP). The NMR experiments were performed in D_2_O at 25 °C and recorded with a Bruker Avance spectrometer operating at 700 MHz for ^1^H, equipped with a 5 mm inverse TXI probe, z-axis gradients, and a Sample Xpress Lite autosampler. The ^1^H-NMR spectra were recorded with a spectral window of 15 ppm, 16 k complex points, and a relaxation delay of 10 s for a total of 16 transients. The ^1^H-^1^H COSY experiments were acquired with spectral windows of 13 × 11 ppm (carrier frequency of 5 ppm) using 2048 × 128 data points, 2 transients, and a relaxation delay of 2 s. The ^1^H-^13^C HSQC experiments were acquired with spectral windows of 13 × 70 ppm (carrier frequencies of 5 and 30 ppm) using 4096 × 56 data points, 4 transients, and a relaxation delay of 2 s. The ^1^H-^1^H TOCSY experiments, implemented with an excitation sculpting scheme for the water suppression [46], were conducted with spectral windows of 20 × 19 ppm (carrier frequency of 4.7 ppm) using 4096 × 256 data points, 24 transients, relaxation delay of 2 s, and a mixing time of 80 ms.

The solubility was determined by preparing a 600 µL solution for each compound in DMSO and buffer solution with a nominal concentration of 400 µM. The effective concentrations were measured at 37 °C using 3-(trimethylsilyl) propionic 2,2,3,3-*d_4_* acid (TSP) as an internal standard.

### 4.5. Glucose Uptake

To evaluate the glucose uptake, both the CAL-27 and MCF-10 A cell lines were seeded at 2 × 10^5^ in 24 plates and incubated overnight at 37 °C. After 24 h, the cells were washed in warm PBS at pH 7.4 and diluted in media without glucose for 40 min. The cells were then washed in PBS and resuspended in suitable complete media and treated with different concentrations of the compounds to assay and incubated with a fluorescent glucose analog (2-deoxy-2-[(7-nitro-2,1,3-benzoxadiazol-4-yl)amino] D-glucose) (2-NBDG) (Sigma-Aldrich) for 1 h at 37 °C. At the end of incubation, the cells were detached through a trypsin solution and PBS-EDTA (without Ca2+ and Mg), washed in PBS, resuspended in FACS flow, and analyzed with flow cytometry through the software Cell Quest (BD Bioscience).

### 4.6. Cell Death and Autophagy

Early RCD-related events were detected through double staining of the cells with fluorescent annexin-V, which preferentially binds phosphatidylserine that appears very early in apoptosis at the external cell surface, and with 7-amino actinomycin D (7-AAD) solution, as a viability dye. The “Annexin V-FITC Kit 7-AAD KiT” (IM3614, Beckman Coulter) was used according to the manufacturer’s instructions. Briefly, 5 × 10^5^ cells were incubated for 15 min with annexin-V-fluorescein isothiocyanate and washed in annexin buffer. Cells were then stained with 7-AAD and analyzed immediately after staining by flow cytometry analysis. The data acquisition and analyses were performed using CytExpert 2.0 (Beckman Coulter, United States) on at least 150,000 events for each sample.

Apoptotic RCD was evaluated in U937 cells by morphological analysis following staining with Hoechst chromatin dye, as previously described [47].

Autophagy was evaluated in the U937 cells using the Autophagy Assay kit from Abcam (ab139484, Abcam, Cambridge, UK) according to the manufacturer’s protocol. In particular, the kit can measure autophagic vacuoles and monitor autophagic flux in live cells using an optimized dye that selectively labels autophagic vacuoles. The 488 nm excitable fluorescent green detection reagent (i.e., green dye) supplied in the kit becomes brightly fluorescent in vesicles produced during autophagy. The nuclear counterstain DAPI (i.e., nuclear dye) is provided in the kit as well to highlight cellular nuclei. The cells were seeded in 24-well plates at a density of 0.5 × 10^6^ cells/mL and treated with vehicle, wortmannin (Sigma-Aldrich W1628), TBT-OCOCF_3_, or a combination of the two for 6 and 18 h. As a positive control, cells were treated for 18 h with the autophagy-inducer rapamycin (Sigma-Aldrich R0395) alone or in combination with the inhibitor wortmannin. At the end of the treatment, samples were collected, washed with 1X assay buffer, and incubated with 100 μL of a dual-detection reagent (1X fluorescent green reagent plus 1X nuclear dye in assay buffer) for 30 min at 37 °C in the dark. Next, the cells were carefully washed three times, and the fluorescence intensity was measured using an appropriate filter set for a fluorescent green reagent (excitation: 463 nm/emission: 534 nm) and nuclear dye (excitation: 350 nm/emission: 461 nm) in a Fluoroskan microplate reader (Thermo Fisher Scientific, Waltham, MA, USA). The autophagic green fluorescence intensity was expressed as the fold change with respect to the control cells using values of the relative fluorescence units (RFUs) measured for green fluorescence normalized to the RFUs measured for the nuclear dye fluorescence in the same sample to control for any change in the number of cells in the samples subjected to different treatments. After washing, aliquots of stained cells were also analyzed by fluorescence microscopy (Leica Leitz DMRE, Wetzlar, Germany). For each sample, images from the same field were taken with a green (for green autophagy-specific reagent) or blue filter (for nuclear stain). Representative fields were photographed using 400× magnification.

### 4.7. Statistical Analysis

The data analysis was performed using the SPSS statistical software system (version 17.0 for Windows, Chicago, IL, USA). The data were assessed using parametric one-way analysis of variance (ANOVA). The statistical significance of the differences among groups were calculated using Bonferroni’s post hoc multiple comparison methods. The results of the statistical tests are reported in the figures and tables.

## 5. Conclusions

In conclusion, the data reported in this study highlight how the final fate of cells exposed to tributyltin compounds is strongly affected by the balance of signals triggering the different forms of RCD and necrosis and of the PI3K/AKT pathway. The elucidation of these aspects could play a key role in the design and synthesis of organotin derivatives alone, and/or in conjugation with a suitable vehicle, or in combination with survival inhibitors to obtain clinically helpful cocktail drugs specifically active towards malignant cells.

## Figures and Tables

**Table 1:** ^1^H and ^13^C chemical shifts of the three tin compounds measured in phosphate buffer (pH 7.4) and DMSO-*d_6_* at 37 °C using TSP as a reference.

Compound			^1^H (ppm)	^1^H (ppm)	^13^C (ppm)
		Position ^1^	Buffer	DMSO	
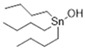	TBT-OH	1	1.210	1.084	19.90
		2	1.558	1.592	29.25
		3	1.323	1.329	28.09
		4	0.901	0.916	15.05
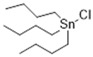	TBT-Cl	1	1.222	1.081	19.94
		2	1.637	1.590	29.45
		3	1.349	1.321	28.19
		4	0.905	0.903	15.33
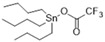	TBT-OCOCF_3_	1	1.164	1.071	19.91
		2	1.617	1.579	29.45
		3	1.343	1.314	28.34
		4	0.899	0.890	15.33

^1^ Position = numbering is sequential and is the same for each compound; 1 refers to tin-bonded hydrogen or carbon up to the terminal methyl represented by position 4.

**Table 2:** Solubility in phosphate buffer and hydrodynamic radii (*R_h_*) of the three tin compounds at 37 °C.

Compound	Solubility (μM)	$R_{h}$ DMSO (Å)	$R_{h}$ Phosphate Buffer (Å)
**TBT-OH/TBTO**	42	5.4	7.8
**TBT-Cl**	61	5.6	13.0
**TBT-OCOCF_3_**	69	5.4	4.6

**Table 3:** Inhibition of the metabolic activity assessed by the MTT assay in CAL-27 cells, MCF-10A cells, PBMCs, and U937 cells treated with the organotin derivatives or cisplatin for 24 h.

	Cells			
Compound	CAL-27	MCF-10A	PBMCs	U937
**TBT-O**	13.18 ± 3.70 ^1^	4.93 ± 2.33	2.96 ± 1.35	1.82 ± 0.21
**TBT-Cl**	0.91 ± 0.53	1.22 ± 0.41	≤1	0.42 ± 0.30
**TBT-OCOF_3_**	2.45 ± 0.14	3.14 ± 2.53	2.68 ± 1.17	1.32 ± 0.32
**Cisplatin**	138.60 ± 59.52	21.50 ± 0.04	52.48 ± 5.88	38.13 ± 3.23

^1^ Data are expressed as the concentration of the compounds needed to inhibit the metabolic activity by 50% (IC_50_) ± SD and refer to three experiments performed in triplicate.

**Table 4:** Effects of TBT-OCOCF_3_ (20 µM) on cell-death-related events in CAL-27 and MCF-10A cells at 4 h after treatment. For the CAL-27 cells, the effects of the cotreatment with wortmannin (WMN) (0.5 µM) were also assessed. Flow cytometry analysis of the cells double-stained with fluorescent annexin-V (Anx) and 7-amino actinomycin D (7-AAD).

Cells	Treatment	Anx-/7AAD-	Anx+/7AAD-	Anx+/7AAD+	Anx-/7AAD+
**CAL-27**	**CTR**	88.01 ± 2.35 ^1^	10.53 ± 1.35	0.06 ± 0.01	0.58 ± 0.02
	**TBT-OCOCF_3_**	86.80 ± 0.01	4.01 ± 0.21	1.26 ± 0.42	7.85 ± 1.01 ^2^
	**WMN**	89.8 ± 4.63	6.42 ± 2.45	0.78 ± 0.04	2.99 ± 0.02
	**WMN+** **TBT-OCOF_3_**	58.8 ± 8.01 ^3^	6.46 ± 0.40	5.56 ± 0.21 ^4^	29.14 ± 3.02 ^3^
**MCF-10**	**CTR**	75.96 ± 5.55	3.68 ± 1.25	9.36 ± 3.24	12.00 ± 2.56
	**TBT-OCOCF_3_**	14.16 ± 2.56 ^5^	24.68 ± 5.45 ^5^	47.59 ± 3.65 ^5^	13.75 ± 1.11

^1^ Data are presented as the mean ± SD from three experiments in duplicate. ^2^ TBT-OCOCF_3_ vs. control, *p* < 0.05. ^3^ WMN+TBT-OCOCF_3_ vs. TBT-OCOCF_3_, *p* < 0.01. ^4^ WMN+TBT-OCOCF_3_ vs. TBT-OCOCF_3_, *p* < 0.05. ^5^ TBT-OCOCF_3_ vs. control, *p* < 0.01.

## Data Availability

Data are contained within the article or Supplementary Materials.

## Supplementary Materials

The following supporting information can be downloaded at: https://www.mdpi.com/article/10.3390/molecules28093856/s1; Figure S1: Regions of the ^1^H-NMR spectra of TBT compounds in DMSO; Figure S2: Viability of U937 cells following treatment with TBT-OCOCF_3_; Figure S3: Selected region of ^1^H-^1^H TOCSY of the 12 bp DNA duplex; Figure S4: Cell cycle analysis of MCF-10A cells treated with TBT-OCOCF_3_.

## Sample Availability

Samples of the compounds are not available from the authors.

## References

[1] Lebwohl D., Canetta R. (1998). Clinical development of platinum complexes in cancer therapy: An historical perspective and an update. Eur. J. Cancer.

[2] Syed Annuar S.N., Kamaludin N.F., Awang N., Chan K.M. (2021). Cellular basis of organotin(iv) derivatives as anticancer metallodrugs: A review. Front. Chem..

[3] Hoch M., Alonso-Azcarate J., Lischick M. (2002). Adsorption behavior of toxic tributyltin to clay-rich sediments under various environmental conditions. Environ. Toxicol. Chem..

[4] Delgado Filho V.S., Lopes P.F., Podratz P.L., Graceli J.B. (2011). Triorganotin as a compound with potential reproductive toxicity in mammals. Braz. J. Med. Biol. Res..

[5] Hobler C., Andrade A.J., Grande S.W., Gericke C., Talsness C.E., Appel K.E., Chahoud I., Grote K. (2010). Sex-dependent aromatase activity in rat offspring after pre- and postnatal exposure to triphenyltin chloride. Toxicology.

[6] Nakanishi T., Nishikawa J., Hiromori Y., Yokoyama H., Koyanagi M., Takasuga S., Ishizaki J., Watanabe M., Isa S., Utoguchi N. (2005). Trialkyltin compounds bind retinoid x receptor to alter human placental endocrine functions. Mol. Endocrinol..

[7] Banti C.N., Hadjikakou S.K., Sismanoglu T., Hadjiliadis N. (2019). Anti-proliferative and antitumor activity of organotin(iv) compounds. An overview of the last decade and future perspectives. J. Inorg. Biochem..

[8] Alama A., Tasso B., Novelli F., Sparatore F. (2009). Organometallic compounds in oncology: Implications of novel organotins as antitumor agents. Drug Discov. Today.

[9] Nakatsu Y., Kotake Y., Takai N., Ohta S. (2010). Involvement of autophagy via mammalian target of rapamycin (mtor) inhibition in tributyltin-induced neuronal cell death. J. Toxicol. Sci..

[10] Katika M.R., Hendriksen P.J.M., van Loveren H., Peijnenburg A. (2011). Exposure of jurkat cells to bis (tri-n-butyltin) oxide (tbto) induces transcriptomics changes indicative for er- and oxidative stress, t cell activation and apoptosis. Toxicol. Appl. Pharm..

[11] Fickova M., Macho L., Brtko J. (2015). A comparison of the effects of tributyltin chloride and triphenyltin chloride on cell proliferation, proapoptotic p53, bax, and antiapoptotic bcl-2 protein levels in human breast cancer mcf-7 cell line. Toxicol. In Vitro.

[12] Basu Baul T.S., Dutta D., Duthie A., Guchhait N., Rocha B.G.M., Guedes da Silva M.F.C., Mokhamatam R.B., Raviprakash N., Manna S.K. (2017). New dibutyltin(iv) ladders: Syntheses, structures and, optimization and evaluation of cytotoxic potential employing a375 (melanoma) and hct116 (colon carcinoma) cell lines in vitro. J. Inorg. Biochem..

[13] Anasamy T., Thy C.K., Lo K.M., Chee C.F., Yeap S.K., Kamalidehghan B., Chung L.Y. (2017). Tribenzyltin carboxylates as anticancer drug candidates: Effect on the cytotoxicity, motility and invasiveness of breast cancer cell lines. Eur. J. Med. Chem..

[14] Waseem D., Butt A.F., Haq I.-u., Bhatti M.H., Khan G.M. (2017). Carboxylate derivatives of tributyltin (iv) complexes as anticancer and antileishmanial agents. DARU J. Pharm. Sci..

[15] Yamada S., Kotake Y., Sekino Y., Kanda Y. (2013). Amp-activated protein kinase-mediated glucose transport as a novel target of tributyltin in human embryonic carcinoma cells. Metallomics.

[16] Bohacova V., Seres M., Pavlikova L., Kontar S., Cagala M., Bobal P., Otevrel J., Brtko J., Sulova Z., Breier A. (2018). Triorganotin derivatives induce cell death effects on l1210 leukemia cells at submicromolar concentrations independently of p-glycoprotein expression. Molecules.

[17] Latsis G.K., Banti C.N., Kourkoumelis N., Papatriantafyllopoulou C., Panagiotou N., Tasiopoulos A., Douvalis A., Kalampounias A.G., Bakas T., Hadjikakou S.K. (2018). Poly organotin acetates against DNA with possible implementation on human breast cancer. Int. J. Mol. Sci..

[18] Pantelic N.D., Zmejkovski B.B., Bozic B., Dojcinovic B., Banjac N.R., Wessjohann L.A., Kaluderovic G.N. (2020). Synthesis, characterization and in vitro biological evaluation of novel organotin(iv) compounds with derivatives of 2-(5-arylidene-2,4-dioxothiazolidin-3-yl)propanoic acid. J. Inorg. Biochem..

[19] Pantelic N.D., Bozic B., Zmejkovski B.B., Banjac N.R., Dojcinovic B., Wessjohann L.A., Kaluderovic G.N. (2021). In vitro evaluation of antiproliferative properties of novel organotin(iv) carboxylate compounds with propanoic acid derivatives on a panel of human cancer cell lines. Molecules.

[20] Attanzio A., Ippolito M., Girasolo M.A., Saiano F., Rotondo A., Rubino S., Mondello L., Capobianco M.L., Sabatino P., Tesoriere L. (2018). Anti-cancer activity of di- and tri-organotin(iv) compounds with d-(+)-galacturonic acid on human tumor cells. J. Inorg. Biochem..

[21] Pellerito C., Emanuele S., Ferrante F., Celesia A., Giuliano M., Fiore T. (2020). Tributyltin(iv) ferulate, a novel synthetic ferulic acid derivative, induces autophagic cell death in colon cancer cells: From chemical synthesis to biochemical effects. J. Inorg. Biochem..

[22] Celesia A., Morana O., Fiore T., Pellerito C., D’Anneo A., Lauricella M., Carlisi D., De Blasio A., Calvaruso G., Giuliano M. (2020). Ros-dependent er stress and autophagy mediate the anti-tumor effects of tributyltin (iv) ferulate in colon cancer cells. Int. J. Mol. Sci..

[23] Waseem D., Khan G.M., Haq I.U., Rashid U., Syed D.N. (2020). The triphenyltin carboxylate derivative triphenylstannyl 2-(benzylcarbamoyl)benzoate impedes prostate cancer progression via modulation of akt/foxo3a signaling. Toxicol. Appl. Pharmacol..

[24] Hubner D., Kaluderovic M.R., Gomez-Ruiz S., Kaluderovic G.N. (2017). Anionic chlorido(triphenyl)tin(iv) bearing n-phthaloylglycinato or 1,2,4-benzenetricarboxylato 1,2-anhydride ligands: Potential cytotoxic and apoptosis-inducing agents against several types of cancer. Chem. Biol. Drug Des..

[25] Zhou M., Feng M., Fu L.L., Ji L.D., Zhao J.S., Xu J. (2016). Toxicogenomic analysis identifies the apoptotic pathway as the main cause of hepatotoxicity induced by tributyltin. Food Chem. Toxicol..

[26] Mitra S., Gera R., Siddiqui W.A., Khandelwal S. (2013). Tributyltin induces oxidative damage, inflammation and apoptosis via disturbance in blood-brain barrier and metal homeostasis in cerebral cortex of rat brain: An in vivo and in vitro study. Toxicology.

[27] Liu W.X., Saito T., Li L., Rinaldi P.L., Hirst R., Halasa A.F., Visintainer J. (2000). Characterization of sn-containing polymer chain ends of polybutadiene using h-1/c-13/sn-119 triple-resonance 3d-nmr. Macromolecules.

[28] Wilkins D.K., Grimshaw S.B., Receveur V., Dobson C.M., Jones J.A., Smith L.J. (1999). Hydrodynamic radii of native and denatured proteins measured by pulse field gradient nmr techniques. Biochemistry.

[29] Schwarzenbach H., Gahan P.B. (2019). Resistance to cis- and carboplatin initiated by epigenetic changes in ovarian cancer patients. Cancer Drug Resist..

[30] Das S., Shukla N., Singh S.S., Kushwaha S., Shrivastava R. (2021). Mechanism of interaction between autophagy and apoptosis in cancer. Apoptosis.

[31] Nakatsu Y., Kotake Y., Komasaka K., Hakozaki H., Taguchi R., Kume T., Akaike A., Ohta S. (2006). Glutamate excitotoxicity is involved in cell death caused by tributyltin in cultured rat cortical neurons. Toxicol. Sci..

[32] Balogova M., Sharma S., Cherek P., Olafsson S.N., Jonsdottir S., Ogmundsdottir H.M., Damodaran K.K. (2022). Cytotoxic effects of halogenated tin phosphinoyldithioformate complexes against several cancer cell lines. Dalton Trans..

[33] Hanahan D., Weinberg R.A. (2011). Hallmarks of cancer: The next generation. Cell.

[34] Ancey P.B., Contat C., Meylan E. (2018). Glucose transporters in cancer—from tumor cells to the tumor microenvironment. FEBS J..

[35] Morselli E., Galluzzi L., Kepp O., Marino G., Michaud M., Vitale I., Maiuri M.C., Kroemer G. (2011). Oncosuppressive functions of autophagy. Antioxid. Redox Signal..

[36] Lim S.M., Mohamad Hanif E.A., Chin S.F. (2021). Is targeting autophagy mechanism in cancer a good approach? The possible double-edge sword effect. Cell Biosci..

[37] Booth L.A., Tavallai S., Hamed H.A., Cruickshanks N., Dent P. (2014). The role of cell signalling in the crosstalk between autophagy and apoptosis. Cell Signal..

[38] Kaku Y., Tsuchiya A., Shimizu T., Tanaka A., Nishizaki T. (2016). Huhs1015 suppresses colonic cancer growth by inducing necrosis and apoptosis in association with mitochondrial damage. Anticancer Res..

[39] Fabrizi C., Pompili E., Somma F., De Vito S., Ciraci V., Artico M., Lenzi P., Fornai F., Fumagalli L. (2017). Lithium limits trimethyltin-induced cytotoxicity and proinflammatory response in microglia without affecting the concurrent autophagy impairment. J. Appl. Toxicol..

[40] Chen S.N., Rehman S.K., Zhang W., Wen A.D., Yao L.B., Zhang J. (2010). Autophagy is a therapeutic target in anticancer drug resistance. BBA-Rev Cancer.

[41] Eberhart K., Oral O., Gozuacik D. (2014). Induction of autophagic cell death by anticancer agents. Autophagy: Cancer, Other Pathologies, Inflammation, Immunity, Infection, and Aging.

[42] Pattingre S., Espert L., Biard-Piechaczyk M., Codogno P. (2008). Regulation of macroautophagy by mtor and beclin 1 complexes. Biochimie.

[43] Petiot A., Ogier-Denis E., Blommaart E.F.C., Meijer A.J., Codogno P. (2000). Distinct classes of phosphatidylinositol 3 ‘-kinases are involved in signaling pathways that control macroautophagy in ht-29 cells. J. Biol. Chem..

[44] Koizumi K., Shintani T., Hayashido Y., Hamada A., Higaki M., Yoshioka Y., Sakamoto A., Yanamoto S., Okamoto T. (2022). Vegf-a promotes the motility of human melanoma cells through the vegfr1-pi3k/akt signaling pathway. In Vitro Cell Dev. Biol. Anim..

[45] Beck J.T., Ismail A., Tolomeo C. (2014). Targeting the phosphatidylinositol 3-kinase (pi3k)/akt/mammalian target of rapamycin (mtor) pathway: An emerging treatment strategy for squamous cell lung carcinoma. Cancer Treat. Rev..

[46] Hwang T.L., Shaka A.J. (1995). Water suppression that works—excitation sculpting using arbitrary wave-forms and pulsed-field gradients. J. Magn. Reson. Ser. A.

[47] Mastino A., Sciortino M.T., Medici M.A., Perri D., Ammendolia M.G., Grelli S., Amici C., Pernice A., Guglielmino S. (1997). Herpes simplex virus 2 causes apoptotic infection in monocytoid cells. Cell Death Differ..

