# Supplementary material for: Biological Evaluation of Triorganotin Derivatives as Potential Anticancer Agents
Source: Molecules. 2023 May 2;28(9):3856. doi: 10.3390/molecules28093856 (PMC10180515; doi:10.3390/molecules28093856)
Supplement: Supplementary file 1 [file molecules-28-03856-s001.zip › molecules-2344138-supplementary.docx]

Supplementary Materials for

**Biological evaluation of triorganotin derivatives as potential anticancer agents**

by Valeria Stefanizzi, Antonella Minutolo, Elena Valletta, Martina Carlini, Franca Cordero, Daniel Oscar Cicero, Erica Pitti, Greta Petrella, Claudia Matteucci, Francesca Marino-Merlo, Antonio Mastino, and Beatrice Macchi

**Contents**

Supplemental Figure S1

Supplemental Figure S2

Supplemental Figure S3

Supplemental Figure S4

**Supplemental Figure S1**

**
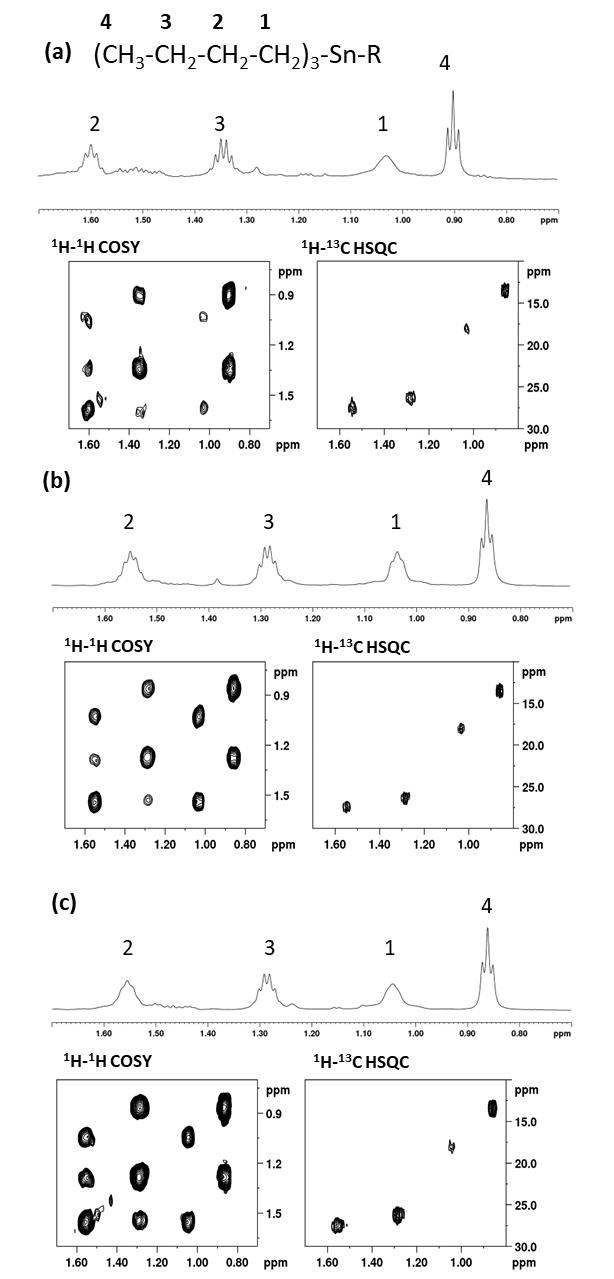
**

**Figure S1:** Regions of the ^1^H-NMR spectra, ^1^H-^1^H COSY, and ^1^H-^13^C HSQC of **(a)** TBT-O (R=OH), **(b)** TBT-OCOCF_3_ (R=OCOCF_3_), and **(c)** TBT-Cl (R=Cl) in DMSO.

**Supplemental Figure S2**


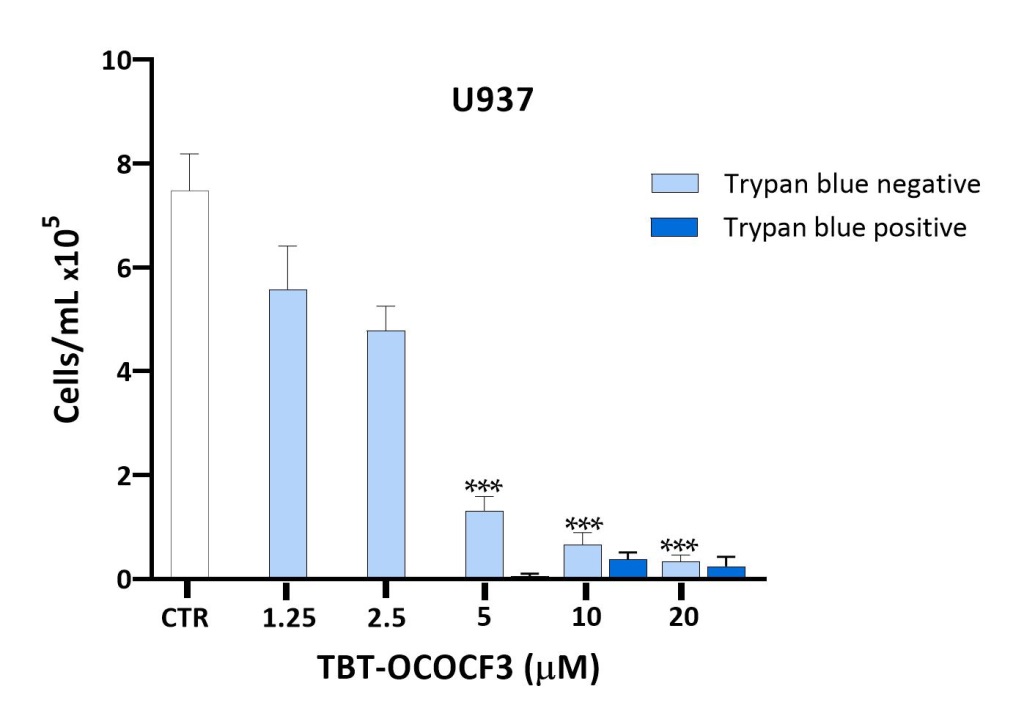


**Figure S2:** Viability of U937 cells following treatment with TBT-OCOCF3. The absolute number of viable and non-viable U937 cells, evaluated as negative or positive cells, respectively, through the trypan blue exclusion test following 24 hours of treatment with TBT-OCOCF3, at concentrations ranging from 1.25 to 20 μM. Data are expressed as mean + S.D. and refer to three experiments performed in triplicate. *** p < 0.001 versus a control group.

**Supplemental Figure S3**

**
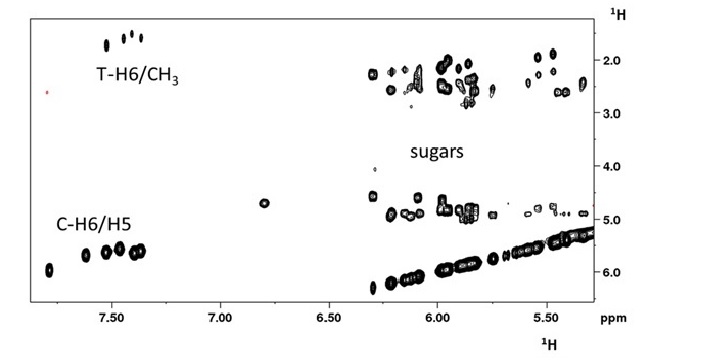
**

**Figure S3:** Selected region of ^1^H-^1^H TOCSY of the 12-bp DNA duplex, showing cross-peaks characteristics of C and T bases and deoxyribose moieties.

**Supplemental Figure S4**

**
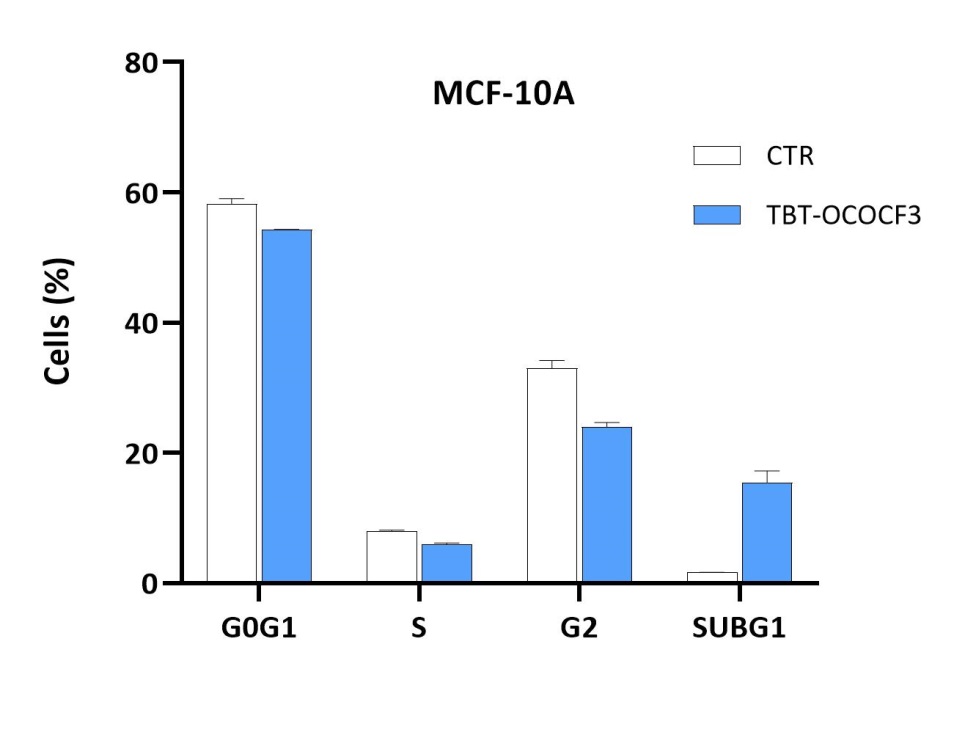
**

**Figure S4:** Cell cycle analysis of MCF-10A cells treated with TBT-OCOCF3. Percentages of cells in the different phases of the cell cycle were assessed in MCF-10A cells treated for 24 hours with TBT-OCOCF3 at 20 μM. Data are expressed as mean + S.D., and refer to three experiments performed in duplicate. Analysis of cell cycle and DNA content by flow cytometry analysis was carried out on samples of cells harvested, washed in PBS, fixed with a mixture of acetone/methanol (1:5, v/v), and stained with 10 μg/ml propidium iodide (PI) plus 0.1 mg/ml RNase (Sigma-Aldrich). Cell cycle phase distribution was measured with a CytoFLEX flow cytometer (Beckman Coulter) and analyzed using CytExpert 2.0 software. G2 and SUBG1 phases, ** p<0.01 TBT-OCOCF3 vs CTR.
